# IntegralVac: A Machine Learning-Based Comprehensive Multivalent Epitope Vaccine Design Method

**DOI:** 10.3390/vaccines10101678

**Published:** 2022-10-08

**Authors:** Sadhana Suri, Sivanesan Dakshanamurthy

**Affiliations:** 1 Dietrich School of Arts and Sciences, University of Pittsburgh, Pittsburgh, PA 15260, USA; 2 Lombardi Comprehensive Cancer Center, Georgetown University Medical Center, Washington, DC 20057, USA

**Keywords:** multivalent epitope vaccine design, immunoinformatics, MHC peptide binding affinity and immunogenicity, deep learning vaccine design, cancer and COVID-19 epitope design

## Abstract

In the growing field of vaccine design for COVID and cancer research, it is essential to predict accurate peptide binding affinity and immunogenicity. We developed a comprehensive machine learning method, ‘IntegralVac,’ by integrating three existing deep learning tools: DeepVacPred, MHCSeqNet, and HemoPI. IntegralVac makes predictions for single and multivalent cancer and COVID-19 epitopes without manually selecting epitope prediction possibilities. We performed several rounds of optimization before integration, then re-trained IntegralVac for multiple datasets. We validated the IntegralVac with 4500 human cancer MHC I peptides obtained from the Immune Epitope Database (IEDB) and with cancer and COVID epitopes previously selected in our laboratory. The other data referenced from existing deep learning tools served as a positive control to ensure successful prediction was possible. As evidenced by increased accuracy and AUC, IntegralVac improved the prediction rate of top-ranked epitopes. We also examined the compatibility between other servers’ clinical checkpoint filters and IntegralVac. This was to ensure that the other servers had a means for predicting additional checkpoint filters that we wanted to implement in IntegralVac. The clinical checkpoint filters, including allergenicity, antigenicity, and toxicity, were used as additional predictors to improve IntegralVac’s prediction accuracy. We generated immunogenicity scores by cross-comparing sequence inputs with each other and determining the overlap between each individual peptide sequence. The IntegralVac increased the immunogenicity prediction accuracy to 90.1% AUC and the binding affinity accuracy to 95.4% compared to the control NetMHCPan server. The IntegralVac opens new avenues for future in silico methods, by building upon established models for continued prediction accuracy improvement.

## 1. Introduction

With the advent of severe acute respiratory syndrome coronavirus 2 (SARS-CoV-2) and the existing cancer burden, the necessity for safe, efficient, and rapid vaccine design is more significant than ever [1]. Due to this urgent need, bio-informatics and immune-informatics have grown when in vitro vaccine testing fails to fulfill the rapid demand, resulting in more significant opportunities for vaccine development. Of these, four methodologies remain the primary options for vaccine design: (1) the classic inactivated viral vaccines, which involve the use of heat/UV radiation-killed tumorigenic components or viral particles that cannot transmit disease; (2) nucleic acid vaccines after sequencing of the entire viral genome; (3) viral vector protein vaccines; and (4) epitopes of viral antigenic proteins used for recombinant peptide subunit vaccines. The advantages of the first three options may include safer preparation and higher concentrations of neutralizing antibodies in the affected area. Ultimately, however, they are unsuitable for immunocompromised patients, who notably comprise a significant portion of the population most affected by COVID. Furthermore, the former three vaccine types often require a large sample body of infectious viruses to succeed, rendering them potentially unfeasible for global-scale development. Thus, epitope vaccines have gained popularity due to their low production costs and decreased allergenicity compared to in vitro vaccine designs [2].

Initially, epitope-based peptide vaccines use pathogenic proteins to design the target peptide’s amino acid sequence. The peptide is then theoretically “produced” when its hypothetical physiochemical properties, such as binding affinity, are determined. The resulting peptides are then linked to form a multi-subunit vaccine evaluated for effectiveness and immunogenicity. These vaccines can be used for tumors as they can instigate the immune system into attacking cancer cells within the body. Vital advantages to this approach include the maintenance of immunogenicity and the ability to survey post-tumor tissue, even long after the tumor has been removed. However, obtaining an adequate number of tumor-reactive T-cells is challenging without immune suppression by a growing tumor, as the potential for autoimmunity allows for damage to subsequent non-tumorigenic tissue. This is because tumors express antigens that may not be specific to the tumorigenic tissue alone. Thus, tumor epitope binding prediction becomes difficult as tumor-associated antigens cannot be determined quickly. Therefore, it is challenging to design in silico prediction models. Moreover, reactions between antigens and surrounding tissue can only be effectively observed in vitro. Observing possible antigenic epitope reactions and side effects can be the difference between life and death for a patient.

Although viral vaccines produce highly neutralized antibodies, cause a high immune response, and allow for easier vaccine design, drawbacks exist. The primary difference between viral vaccines, DNA and mRNA vaccines, involves the antigen coding with plasmid DNA. Expressing plasmid DNA in concentrated quantities may eventually result in high toxicity, and, thus, repeated doses would be unwise. In the current pandemic climate, such a restriction may do more harm than good as variants and mutations in the virus continue to be discovered [3]. This contributed to our rationale for investigating epitope vaccine design with the generation of peptide binding predictions instead, using AI and deep learning methods. A primary advantage of deep learning is the ability to organize data to simulate vaccine efficiency for different peptide sequences. The resulting binding affinity predictions can then be attributed to B and T cell epitopes due to their cellular and humoral responses [2].

Such in silico tools have revolutionized many scientific fields, ranging from immunochemistry to molecular genetics. Over one million proteins are thought to exist on a cell’s surface, to be available for binding to T-cells [4]. Such patterns would be near impossible to replicate by human trial—the lack of a single universal vaccine for a disease suggests the necessity of testing a myriad of antigen and epitope-differentiating strategies for effective treatment. Thus, computational methods allowed us to compare large amounts of proteins to produce viable data while optimizing the chances of successful prediction. Compared to experimental methods, in silico tools decrease the time and costs necessary for accurate prediction.

Still, drawbacks exist for in silico methods for vaccine design: the existing methods automatically compute dozens of subunit locations for choosing epitopes while only 10–20 are needed. An overly large dataset to select from hinders the eventual manual process of subunit selection as much of the produced data may be irrelevant. The servers used for our study were, therefore, chosen to address these limitations. For example, the DeepVacPred server (https://github.com/zikunyang/DCVST, accessed on 15 May 2021) creates an output of 30 subunit candidates at most to prevent excess data production. This model was retrained in IntegralVac to predict even fewer candidates depending on the immunogenicity scores of the input peptide sequences. Likewise, the MHCSeqNet tool refines binding affinity predictions for MHC class I molecules, and the Hemolytic model allows expansion of predictions to peptide sequences from non-viral sources. We referenced both tools in continuing to expand the coverage of IntegralVac. In developing our method, we noted that two features are most important for vaccine design: ensuring that the peptides have immunity specific to multiple antigen types and increasing the inducibility in the immune system. Our study successfully increased prediction accuracy compared to the NetMHCPan server control by integrating the three methods addressing antigenicity and immunogenicity.

## 2. Materials and Methods

### 2.1. Data Collection and Selection

IntegralVac’s initial validation was performed with approximately 4500 experimental peptides from the IEDB database. These peptides were selected using the IEDB’s automated MHC Class I prediction dataset (http://tools.immuneepitope.org/main/datasets; accessed on 15 May 2021), covering over 160 MHC molecules for peptides of lengths 8 to 10. These data were further subdivided into sections for cross-validation to prevent a repetition of 8 mer segments between each validation run. Next, for the second round of cross-validation, only MHC Class I alleles with a frequency greater than 1% were selected and sorted by percentile rank and IC_50_ values (Supplementary Tables S1–S8). After data collection, we began to optimize raw NetMHCPan reference code and made predictions for in-house retrieved variant sequences. These sequences included CD8+ T-cell epitopes of SARS-CoV-2 nsp3 and nsp12, ORF3a, and ORF9b variants. Probable non-allergenic and non-toxic epitopes were then selected using allergenicity and antigenicity checkpoint filters, as referenced from the DeepVacPred model. We continued to use NetMHCPan as the control method for making predictions, as it is the established method trained on both binding affinity and ligand data. Thus, it contains the widest coverage of both MHC Class peptides; all three of our referenced models likewise used NetMHCPan as the primary mode of comparison against their performances [1].

### 2.2. Machine Learning Model Selection for IntegralVac

The DeepVacPred model is a vaccine subunit prediction framework that selects immunogenic peptides as potential vaccine subunit candidates. It selects immunogenic fragments within larger viral proteins and combines them to create a vaccine against that viral strain. NetMHCPan does construct a predictive vaccine model as the DeepVacPred server did but instead stops identifying immunogenic proteins from the complete peptide sequences. However, DeepVacPred uses more checkpoint filters than NetMHCPan to predict immunogenic fragments in peptide sequences so that DeepVacPred can generate more accurate predictions than NetMHCPan. Thus, as we aim to increase immunogenicity prediction accuracy in our model, we chose to reference DeepVacPred methods for improving the accuracy of peptide immunogenicity score predictions compared with NetMHCPan (https://services.healthtech.dtu.dk/service.php?NetMHCpan-4.1, accessed on 15 May 2021). 

Along with immunogenicity predictions, we also pursued peptide binding affinity prediction capability. Although DeepVacPred uses the NetMHCPan server to generate affinity predictions for Class I CTL epitopes, we only generated affinity predictions for MHC I epitopes. Thus, we did not reference DeepVacPred for the next phase of our study. Instead, we next examined the server MHCSeqNet. The MHCSeqNet server predicts the binding affinity of peptides solely based on their amino acid sequence, regardless of their MHC class type. This was advantageous as a reference model as it allowed us the possibility of future improvements, such as expanding IntegralVac’s coverage to multiple MHC classes instead of only MHC I epitopes. We wanted all other data to remain as consistent as possible with experimentally verified tumor data and, thus, needed a server that could predict for IEDB MHC I peptides. Unlike the control NetMHCPan server, MHCSeqNet can handle variable amino acid sequence length. We handled entire peptide sequences despite examining the amino acid structure much like the DeepVacPred server. On the other hand, MHCSeqNet uses an amino acid embedding model rather than a protein-encoding system that uses an entire peptide sequence as a single input. Thus, we integrated new methods so that IntegralVac could simultaneously use both an entire peptide sequence and individual amino acid properties within a sequence as dual inputs. We also re-trained the models for different datasets by integrating the MHCSeqNet model with the aforementioned DeepVacPred.

Finally, we used the outlier detection method of Winsorization. As we were using such large datasets from multiple sources, we wanted to ensure that IntegralVac could successfully make predictions without being skewed by outliers by optimizing the area under the curve (AUC). In doing so, we referenced HemoPI, a tool investigating non-hemolytic peptides that assessed multivariate outlier detection methods to predict hemolytic toxicity values for selected peptides. We did not use hemolytic toxicity as a checkpoint filter for IEDB-obtained data; instead, we only used the prediction tool ToxinPred for in-house toxicity predictions. Despite this, we still referenced the non-hemolytic peptide model to help us optimize and remove outliers from our previously integrated models. In conjunction, the three methods allowed for a more comprehensive tool for predicting peptide properties with greater accuracy than the control NetMHCPan. Each referenced model was selected for its high accuracy in predicting epitope immunogenicity of various datasets.

### 2.3. Comparison of IntegralVac MHC 1 Peptide Prediction with the NetMHCPan

**Figure 1:**
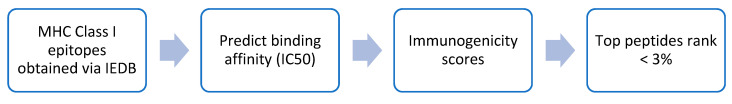
IntegralVac’s MHC Class I data selection process workflow. IntegralVac initially obtains epitopes using the IEDB analysis and search tool. The epitopes are then used as input in IntegralVac to generate binding predictions (IC50 values) for each peptide. We generate immunogenicity scores for all peptides using predictor characteristics of the peptide that may include amino acid sequence, antigenicity, allergenicity, and toxicity values. Most top peptides are selected based on their near or below 3% percentile ranking values. After selection, IntegralVac was utilized to compare the top-ranking peptides with results from the NetMHCPan EL server. Unlike the other datasets, MHC Class I data did not use each peptide’s antigenicity, allergenicity, and toxicity values as predictors.

We first tested IntegralVac on IEDB MHC Class I epitopes using the NetMHCPan EL 4.1 server as a control. Additionally, we also obtained the experimental epitopes from the IEDB epitope resource. The epitopes were ordered by three metrics. The first metric is a binding affinity to denote IC50 values. Although the server’s binding affinity values are natural log functions of each epitope’s IC50 value (pIC_50_), we represented this value through the actual IC50 values to show binding potency. The second metric is the percentile ranking of each peptide compared to a randomly generated epitope within the IEDB database. Lower percentile values indicate higher affinity, and we designated epitopes with lower than 3% percentile rankings as the top predicted epitopes (Supplementary Table S9). The third metric is enrichment score, a log function of a peptide’s amino acid ratio in an immunogenic dataset to a non-immunogenic dataset. This last metric is directly related to the peptide’s overall immunogenicity score for all peptides. Along with these measures, we modified both the DeepVacPred and MHCSeqNet models with additional rounds of Winsorization to optimize predictions of T-cell CD8+ epitopes (Figure 1).

### 2.4. Increasing Coverage of IntegralVac Using CTL Epitopes from the DeepVacPred Model

Our next aim was to increase the accuracy and AUC of IntegralVac’s predictions for immunogenicity and begin testing IntegralVac on larger datasets. The DeepVacPred server was used as a reference in predicting binding affinities for linear B-cell epitopes, Cytotoxic T Lymphocytes (CTL) epitopes, and Helper T Lymphocytes (HTL) epitopes. Despite its capability to process larger datasets, DeepVacPred had limited data coverage with respect to the tumor peptides we later used in our study because DeepVacPred cross-linked only one B-cell epitope and one T-cell epitope for data validation. Thus, DeepVacPred may not have produced as sufficiently high AUC and accuracy values as it could have due to data limitations. The discrimination threshold was repeatedly lowered to define more extensive target epitope data until eventually stabilizing at the low value of 0.32. Lower threshold values indicate that a DNN server has a weaker classification ability, limiting a prediction tool’s potential validity. 

**Figure 2:**
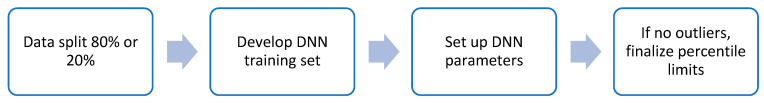
Visualization of the steps needed for the Winsorization process of MHC Class I epitopes. We first split the data using an 80–20 ratio to set up a training set. This was followed by developing the DNN with the same number of Hidden Layers corresponding to the amount of reference data. We used an Adam algorithm and a Xavier initialization method to set up the DNN parameters, including learning rate, optimizers, epochs, and batch size. This process was repeated until all outliers within the selected range were eliminated. Once no outliers were present, we continued training the DNN by finalizing the upper and lower percentile fences. We used these same steps for in-house datasets, albeit with a 90:10 training-to-test ratio instead of 80:20.

We utilized an additional three rounds of cross-validation to address these limitations due to the larger datasets used for this study. This finalized the test-to-train ratio as 90:10 as opposed to the default 80:20 ratio, which was used for the MHC Class I dataset. This also addressed the possibility of an error during integration that arises due to interference from previously integrated models, by reducing representational overlap within our datasets, such as regions of sequence similarity between sequences (Figure 2). For example, intermittent rounds of cross-validation between the integration of MHCSeqNet and HemoPI helped to provide a “clean slate” for HemoPI to run its own prediction algorithm without the worry of MHCSeqNet skewing its accuracy in either direction. Moreover, we chose to integrate methods from each model that differed in function within the prediction framework, so that the two models would not compete for more accurate predictions. We then performed three rounds of optimization using the existing three optimizer tools coupled with Winsorization [5] or the replacement of data outliers with more valid data points instead of truncating existing data entirely. The parameters for this Winsorization were limited to the 10th percentile of lowest-value outliers and 20% of greatest-value outliers as part of mini-batch learning. We subsequently used hydrophilicity, exposed surface area, amino acid sequence, and polarity properties to enhance prediction accuracy [6], along with the physiochemical properties used by the NetMHCPan server. Immunogenicity was calculated using the amino acid properties of each peptide and, therefore, used as a positive control as it was projected to remain unchanged between the NetMHCPan server and our deep learning tool. 

The hyperparameter values of the DNN training framework are listed below. The objective was to maintain a high computing speed by optimizing the different parameters while maintaining high accuracy.

- Learning rate: [0.0001, 0.001, 0.002];
- Optimizers: [SGD, RMSProp, Adam];
- Epochs: [2000, 4000, 6000, 8000, 10,000];
- Batch size: [512, 1024, 2048, 4096]. [7]

**Figure 3:**
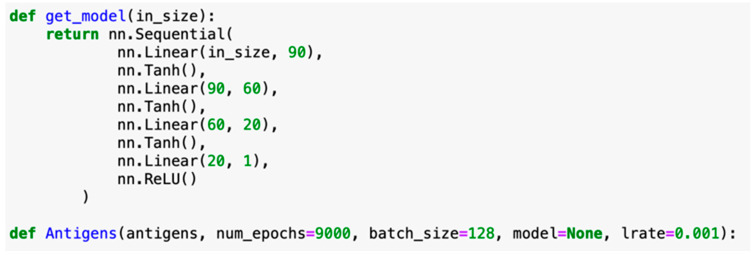
Learning rate adjustment code based on DeepVacPred parameters. Reprinted/adapted with permission from Ref. [7]. 2021, Yang, Z., Bogdan, P., & Nazarian, S. [7] (Supplementary File S10).

Learning rate as a hyperparameter was utilized to determine how much variation should exist in the model in the case of any data parsing error, which often affected the program runtime. As the DeepVacPred server used a learning rate value of 0.001 and had an efficient program runtime, we also used 0.001 instead of the default learning rate of 0.01. The number of epochs, denoting the number of iterations run through an entire dataset, should increase with lower learning rates as the data must be further parsed through the network. We alternated between 6000 and 8000 epochs depending on whether we used an IEDB dataset versus a smaller in-house dataset. In the case of a smaller dataset, we kept the batch size constant to randomize the selection of peptides regardless of dataset variation (Figure 3).

### 2.5. Training IntegralVac for Multiple Known MHC Sequence Datasets with MHCSeqNet 

**Figure 4:**
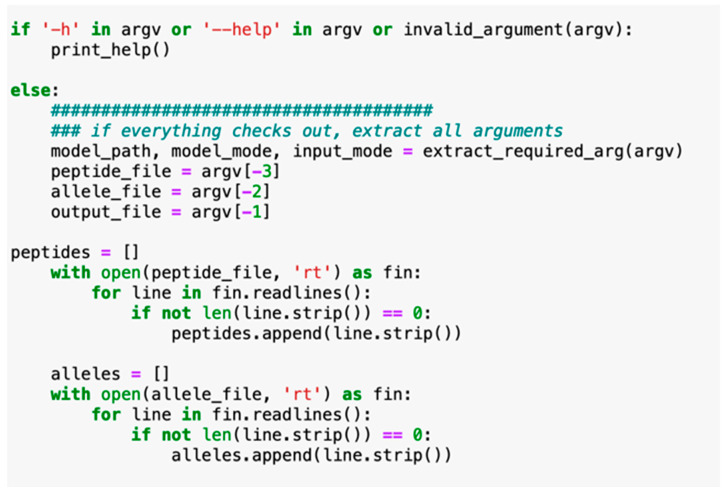
IntegralVac used the code for retraining to support the input of multiple peptide lengths and types, as opposed to Swiss-Prot database proteins and peptides of amino acid length 9 [8]. We instead used peptides of lengths 7–9 depending on the dataset.

Our next aim for IntegralVac was to generate binding affinity predictions for novel MHC allele sequences that were not previously specified in the training dataset [8]. We utilized the MHCSeqNet tool, which involved two inputs, the peptide sequences and the MHC allele for the human peptides, to be prepared through an embedding layer and an additional processing layer. Both inputs are then passed through a final classification layer before generating an output [8]. In re-writing MHCSeqNet with Gated Recurrent Units (GRUs), we restricted the evaluation sets to 40 MHC alleles (for the default NetMHCPan) to support input peptides of any length (Figure 4). We have included the complete code for the referenced methods with licensed use from the authors in the Supplementary Materials (Supplementary File S11).

### 2.6. Determining Peptide Characteristics in IntegralVac Using HemoPI Peptide Vaccine Methods

**Figure 5:**
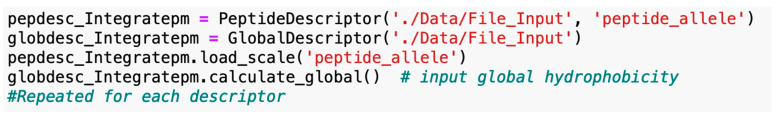
We utilized the input portion from the non-hemolytic peptide model’s descriptor [9]. We aimed to run through a list of peptide allele descriptors specified by the program, which were hydrophobicity values for the original code. The complete code for the referenced methods, as allowed for use by the HemoPI creators’ license, is in the Supplementary Materials (Supplementary File S10).

We next examined a non-hemolytic peptide model in peptide characteristic determination [9]. Although we were not investigating the hemolytic properties of the peptide data used for IntegralVac, we still wanted to examine the structural properties of the dataset to be used as possible additional predictors as part of IntegralVac. For reference, we have displayed an excerpt of HemoPI’s method for determining hydrophobicity descriptor properties for each peptide in Figure 5. Although we did not use hydrophobicity as a descriptor, we utilized similar methods in examining the amino acid structures of each peptide.

The non-hemolytic peptide model predicted hemolytic activity with 95–97% accuracy through gradient boosting classifiers, i.e., producing a more robust prediction model by combining several weaker learning models. IntegralVac utilized several additional binary classifier methods, including Logistic Regression LOGREG28, Linear and Quadratic Discriminant Analysis LDA/QDA30, Support Vector Classifier SVC (with the four kernels: linear, radial basis function, polynomial, and sigmoid) 31, and Adaptive Boosting Classifier ABC to improve prediction accuracy [9]. The hemolytic peptide model split its datasets into the more significant training set (80%) and a smaller test set (20%) for external validation. Still, we chose to keep the 90:10 ratio for consistency throughout the study. The resulting regression model was loosely constructed to confirm the Winsorization of error outliers. We used IntegralVac to simulate predictions upon completing the updated regression model, using NetMHCPan as the baseline comparison. After successfully integrating the three models, a new ROC curve was generated for the finalized model.

### 2.7. Antigenicity and Allergenicity Prediction Tool Selection

Prediction of peptide properties by directly comparing sequences is advantageous for its speed and cost-efficiency. However, this prediction method can pose obstacles in determining more abstract peptide characteristics, such as antigenicity and allergenicity. For example, some proteins may have similar structural properties but still do not have easily identifiable similarities in their sequences because antigenicity can be encoded in a myriad of ways for a single class of proteins. Thus, instead of directly comparing sequence alignments for antigenicity predictions, we relied on the Vaxijen server, which uses auto cross-covariance methods. These methods transform protein sequences into amino acid property vectors [10]. The AntigenPro server similarly does not rely on similarities in amino acid positions for antigenicity predictions but still uses sequence, unlike the Vaxijen server. It computes features directly related to the sequence, such as length and residue charge, and uses these values to obtain predicted antigenicity features. Finally, the AllergenFP server also uses auto cross-covariance methods, such as the Vaxijen server for allergenicity. It is a binary classifier for allergens and non-allergens after transforming the peptides into uniform vectors [7].

## 3. Results

### 3.1. The IntegralVac Method

**Figure 6:**
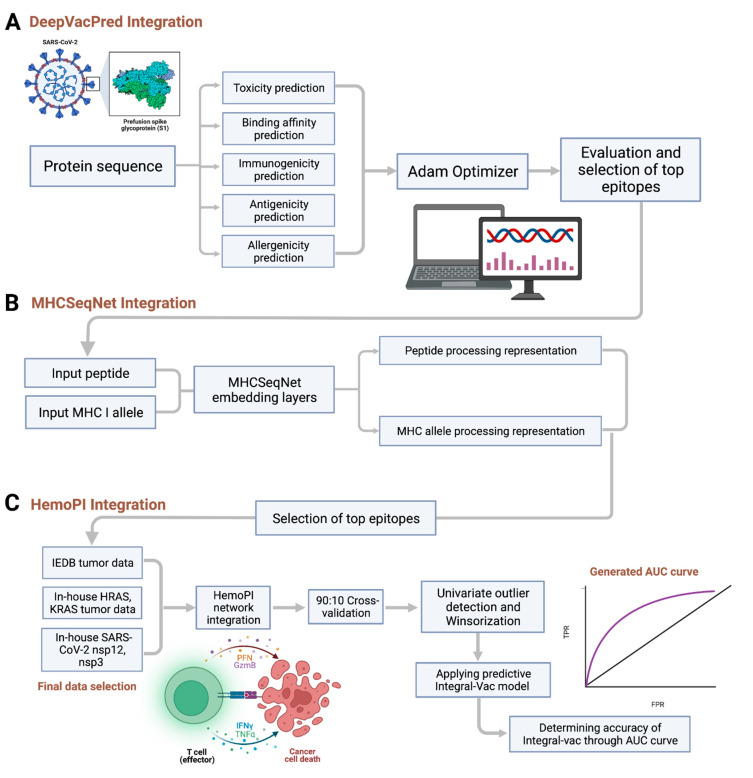
The overall workflow of the IntegralVac. The IntegralVac method integrates the DeepVacPred, MHCSeqNet, and HemoPI models in that order. Our method operates by initially predicting binding affinity, then immunogenicity scores, and then use of optimization to ensure that outliers are excluded from the amino acid sequence data used for making these predictions. Finally, other checkpoint filters are also predicted, and final rounds of optimization are performed. (**A**) describes the initial integration of DeepVacPred into DeepVacPred using the Adam optimizer. As per (**A**), IntegralVac utilizes DeepVacPred to expand upon binding affinity and immunogenicity predictions with additional checkpoint prediction filters, including toxicity, antigenicity, and allergenicity, followed by optimization. Part (**B**) involved using MHCSeqNet’s embedding layers to further optimize the model for MHC Class I peptides. This method helped us remove duplicated and ambiguous entries from IntegralVac’s dataset, and the Adam optimizer in the previous step also removed low-confidence entries. This exclusion of outlier data contributed to slightly improving prediction accuracy. Following MHCSeqNet integration and further narrowing down the list of top epitopes, (**C**) indicates HemoPI integration along with finalized optimization of IntegralVac. Additionally, these epitopes were combined with in-house cancer and COVID dataset, then cross-validated and Winsorized. Together, the integrated models make up IntegralVac, of which the AUC and accuracy were measured.

The entire workflow for IntegralVac development is shown in Figure 6. The primary goal of IntegralVac is to improve selected epitopes’ binding affinity and immunogenicity predictions, ultimately for better vaccine design. We used the IEDB and DeepVacPred servers as a reference for doing so. IntegralVac began with the initial modification to the established NetMHCPan methods for predicting solely binding affinity. IntegralVac then referenced DeepVacPred two-fold, combining clinical checkpoint filters and individual amino acid sequences for each peptide across all datasets to improve prediction accuracy with additional predictors. IntegralVac optimized these predictors to increase the accuracy of the overall scoring of the HLA peptides against the top HLA alleles.

#### 3.1.1. DeepVacPred Integration

We used the IEDB primarily in two ways: first, the IEDB’s NetMHCPan prediction tool was used to make binding affinity predictions. Second, we confirmed the accuracy of IntegralVac for multiple checkpoint filter variables, including allergenicity, antigenicity, and toxicity. They were implemented on experimental data obtained from the IEDB that included epitopes selected from the top HLA alleles globally, i.e., the alleles with the greatest population coverage. When obtaining immunogenicity scores, each checkpoint filter was predicted using the Vaxijen, AllergenFP, AntigenPro, and ToxinPred prediction servers.

DeepVacPred also predicted values for similar checkpoint filters to IntegralVac, albeit with different servers, such as the AllerTOP server for allergenicity. However, we chose to keep the same predictor tools, i.e., AllergenFP, that we had previously used for experimentally tested in-house data, including pancreatic cancer peptides (HRAS and KRAS) and non-structural COVID. This is because we had already verified that these tools were compatible with our optimization code using Adam and other Winsorization methods [8]. This was coupled with integrating other methods, i.e., MHCSeqNet, to simultaneously improve the accuracy of IntegralVac and validate IntegralVac for multiple datasets. DeepVacPred was the primary reference model for IntegralVac and required more detailed steps compared to the other models MHCSeqNet and HemoPI. This integration of DeepVacPred is expanded upon in Section 3.2.

#### 3.1.2. MHCSeqNet Integration

IntegralVac used the MHCSeqNet method as a reference to begin examining the properties of individual amino acid sequences for each peptide as opposed to the entire peptide sequence as a single unit. Thus, information from the amino acid sequence of each input peptide was integrated with other checkpoint filters to generate immunogenicity score predictions for each peptide. This was then depicted by a percentile ranking (Supplementary Tables S1–S8). IntegralVac conducted intermittent rounds of optimization to validate the method within this process.

#### 3.1.3. HemoPI Integration

Having obtained the final dataset for selecting top epitopes, we carried out cross-validation with the integration of HemoPI, following amino acid sequence integration with checkpoint filters to predict epitope immunogenicity and binding affinity. We used the SVM-based solubility prediction tool SolPro as a reference for further cross-validation within IntegralVac, albeit for immunogenicity and not solubility. SolPro predicts protein solubility with 10-fold cross-validation; we instead used three-fold cross-validation at window length 16 with a threshold value of 0.32 for our DNN [7]. Finally, we again optimized the overall method using the Adam optimization algorithm. We did not immediately assess the accuracy of IntegralVac after implementing these steps to validate our method, as we decided to further test IntegralVac on other datasets before validating our method with graphical analysis. Our rationale for doing so was that our primary reference model, DeepVacPred, also tested its framework on several datasets before checking for accuracy. Similarly, we aimed to expand IntegralVac’s scope to viral and tumor data to uncover the peptide vaccine predictor’s full potential.

### 3.2. Integration of DeepVacPred into IntegralVac: Immunogenicity Predictions with Clinical Checkpoint Filters

We primarily utilized two viral datasets, DeepVacPred, and in-house SARS-CoV-2 data, to validate IntegralVac. Our first aim was to ensure that IntegralVac’s results would be consistent with DeepVacPred even after the modifications for using checkpoint filters made by integrating other deep learning tools so that we could continue to improve accuracy for other types of datasets. This was completed via the clinical checkpoint filters used in the DeepVacPred server. The DeepVacPred server predicts cytotoxic T lymphocytes (CTL) epitopes using MHC Class I T-cell HLA alleles. We validated IntegralVac using the DeepVacPred server’s top generated vaccine peptide subunits to predict HLA alleles and their supertypes. Using all epitopes and their associated alleles as input, we modified the DeepVacPred code with repeated optimization to increase immunogenicity ranking accuracy and later integrate the checkpoint filters.

The primary goal of the DeepVacPred server was not to predict individual peptide sequence immunogenicity scores. Instead, it aimed to construct a complete multi-subunit vaccine, and the server simply generated immunogenicity rankings and binding affinity predictions in the process. Regardless, we chose to reference the tool despite IntegralVac stopping at immunogenicity and binding affinity predictions without constructing a full in silico vaccine because DeepVacPred’s near-perfect accuracy and high validity compared with our control IEDB tools. Thus, we then integrated modifications from other tools as necessary to improve upon the peptide prediction aspects of DeepVacPred for our purposes. Despite our modifications, our results matched the DeepVacPred server’s predicted sequences. Predictions were made using the most common 12 HLA Class I alleles cited by the DeepVacPred model: HLA-A1, HLA-A2, HLA-A3, HLA-A24, HLA-A26, HLA-B7, HLA-B8, HLA-B27, HLA-B39, HLA-B44, HLA-B58, and HLA-B62 [7]. These 12 alleles were tested for overlapping peptide sequences (sequence length 9) to make homogeneous CTL epitopes predictions for solely DeepVacPred peptides. Like the DeepVacPred model, we also used the Vaxijen, AllergenFP, AntigenPro, and ToxinPred prediction servers to assess antigenicity and allergenicity and ensure that IntegralVac did not affect the original server’s ability to use antigenicity, allergenicity, and toxicity as prediction checkpoint filters. Here, our purpose is to measure antigenicity to ensure consistency with the DeepVacPred server code, thus rated with the assumption that Vaxijen and AntigenPro were equally accurate antigenicity prediction servers. The top five epitopes were obtained and cross-referenced with the DeepVacPred server’s predictions (Table 1).

### 3.3. Human Cancer and COVID Epitope Data Predictions from In-House Data Sets

After initial optimization, we aimed to expand the coverage of IntegralVac for a multitude of datasets, including both tumor peptides and COVID epitopes. We used in-house published data of the HRAS proto-oncogene for tumor peptides that were investigated as mutations in the genes associated with cancer development [10]. This included our earlier HRAS dataset that was generated by obtaining the unmutated HRAS peptide sequence from the National Library of Medicine, then using the top 15 HRAS mutations (G12C, G12D, G12S, G12V, G13C, G13D, G13R, G13S, G13V, A59T, Q61H, Q61L, Q61R, and E62G) to produce 44 mutated sequences [10]. Next, epitopes from the IEDB server were retrieved using the filters: human epitope source, T-cell assays (negative or positive), MHC Class I restrictions, human hosts, cancer epitopes only, and linear peptides of length 8–10. For viral data, we first referenced the DeepVacPred model’s dataset consisting of known T-cell B-cell epitopes retrieved using the accession number MN908947 from the NCBI database [7]. We then used isolated sequences from non-structural SARS-CoV-2 and non-structural protein MHC I COVID epitopes. This included CD8+ SARS-CoV-2 sequences obtained from the NCBI database, with accession number NC_045512. Specific protein accession numbers were used for each multi-domain protein: “YP_009725299.1 (NSP3), NCBI: YP_009725307.1 (NSP12), NCBI: YP_009724391.1 (ORF3a) and UniProtKB/Swiss-Prot: P0DTD2.1 (ORF9b)” [11]. The peptides with a frequency greater than 1% were selected for further processing for all datasets.

### 3.4. Generating Amino Acid Predictor-Based Immunogenicity Rankings

IntegralVac measured similarities and differences in amino acid sequence and structure for each peptide sequence to generate immunogenicity scores. These predicted scores were used to rank peptides. As IntegralVac had generated predictions for IEDB Class I peptides with greater accuracy, we wanted to verify whether IntegralVac’s immunogenicity rankings would also change compared to NetMHCPan. We began by comparing two peptide datasets: the first included data selected from the DeepVacPred, and the second had data selected from the curated IEDB protein sequence database. The resulting comparisons, along with IC50 scores and binding affinities, were used to obtain percentile rankings for each compared peptide, ordered by similarity. Lower percentile rankings for a given peptide indicate higher binding affinity. The resulting rankings provided a consistent measure of comparison and scaling that could be used across all datasets and were efficient for using recurring neural networks (RNNs). In our results, the peptide rankings and binding affinities stayed consistent between all datasets compared to NetMHCPan values. Still, we were able to expand predictions to a greater range of data than the original, including tumor data, thus validating the accuracy of IntegralVac. Likewise, there was no significant difference in immunogenicity scores of our datasets compared to those predicted by the NetMHCPan server. Thus, IntegralVac produced reliable results for Class I epitope data in the range of 2500–5000 peptides per dataset.

The top epitope rankings generated by IntegralVac did not differ from the rankings of the IEDB NetMhcPan server, so we must assume that the control immunogenicity rankings already had high accuracy. Prediction accuracy increased approximately 1.6%, and AUC increased 5.2% compared with the NetMHCPan server. Upon successful predictions of the individual peptide sequences that we used as an initial test, we began binding affinity and immunogenicity predictions for in-house data [10]. This included peptide data obtained from KRAS, HRAS, murine ORF, and SARS-CoV-2 sequences and was used previously in our in-house epitope selection tools using the IEDB server [11]. These sequences made the ideal first target to verify whether IntegralVac could simultaneously generate predictions for multiple heterogeneous datasets (i.e., data from various populations). Moreover, our study also generated antigenicity, allergenicity, and toxicity predictions for these datasets, as described later. We made successful predictions for each CD8+ epitope data-set for SARS-CoV-2 and cancer datasets obtained from referenced deep learning models and the IEDB database (Table 2).

### 3.5. Immunogenicity Predictions for Coronavirus Data

Wild-type and transgenic murine proteins provide relevant models that can mimic human immunogenic responses provided the cell line has appropriate stimulation. Thus, we aimed to examine predicted murine variant-induced responses. We used the murine datasets that we investigated in our previous studies, the experimental sequences with strong affinities for murine MHC restriction [11]. In particular, mouse ORF proteins are viral proteins that are highly conserved within the coronavirus, and two non-structural proteins (nsp), were investigated in particular for the integrated model: nsp12 (Supplementary Table S3) and nsp3 (Supplementary Table S4). ORF protein sequences were also analyzed as part of larger datasets. IntegralVac used ORF3a (Supplementary Table S5) and ORF9b (Supplementary Table S6) data, as both were hypothesized to be effective vaccine targets due to their multi-allelic binding abilities that induce immunogenicity. Finally, N-protein (Supplementary Table S7) and S-protein data for SARS-CoV-2 (Supplementary Table S8) were used as input. The top peptides generated for each dataset remained consistent with those predicted by the IEDB database. The primary differences in our data compared with experimental IEDB data were sequence length and worldwide population coverage, yet verifiable results were produced for both. Thus, despite not being clinically experimented with (i.e., as IEDB peptides were), we could use in-house data as accurate prediction comparisons and verify our results using the IEDB MHC Class I analysis tool.

### 3.6. Validation of Integral-Vac with KRAS/HRAS Tumor Epitopes

We next investigated the cancer peptides using the IEDB experimental peptide data to determine IntegralVac’s relevance to both viral and tumor data. This was considered especially relevant because the field of AI-based peptide vaccines continues to expand to cancer research. In doing so, we first selected all our study’s MHC class I human cancer epitopes from the IEDB database for a final round of predictions (Supplementary Table S9). As the IEDB server is continually updated to generate a comprehensive source of experimentally tested epitope data, we attempt to improve the IEDB data predictions and verify IntegralVac’s validity for a range of datasets. This included both the predicted epitopes (as was done referencing the DeepVacPred server) and existing clinical epitope data. We did not analyze population coverage in this study; instead, we used the IEDB population coverage analysis tool to determine worldwide human population coverage for in-house data [11]. We predicted binding affinity and immunogenicity scores using the same filtration methods as the IEDB’s NetMHCPan server. Though the immunogenicity rankings varied between the control NetMHCPan predictor and our study’s integrated model, overall binding scores remained consistent for the tested data (Supplementary Table S1–S8), thus validating IntegralVac.

As pancreatic tumors remain particularly resistant to chemotherapy, vaccine design is of great interest in developing future treatment methods [12]. Our previous study analyzed KRAS murine peptides for their implications in pancreatic cancer [10]. Although our top peptides were the same as IEDB predictions for immunogenicity scores and percentile rank order, IntegralVac was able to make predictions for a larger dataset of variable peptide lengths with fewer prediction filters (Supplementary Table S1). We found two overlapping epitopes between unmutated and mutated datasets. Likewise, we made predictions with the same accuracy as IEDB for HRAS murine epitopes but with fewer checkpoint filters necessary [10]. We then predicted a more significant number of peptides than the KRAS epitopes but with the same relative diversity (Supplementary Table S2). All mutated HRAS epitopes were selected from literature reviews of the HRAS gene. Before validation with Integral-Vac, these epitopes were validated by us previously using the NetH2Pan to predict binding affinity [10]. We selected the top epitopes from this dataset upon completing affinity predictions.

### 3.7. Validation of IntegralVac Predictions Using ROC Curve

**Figure 7:**
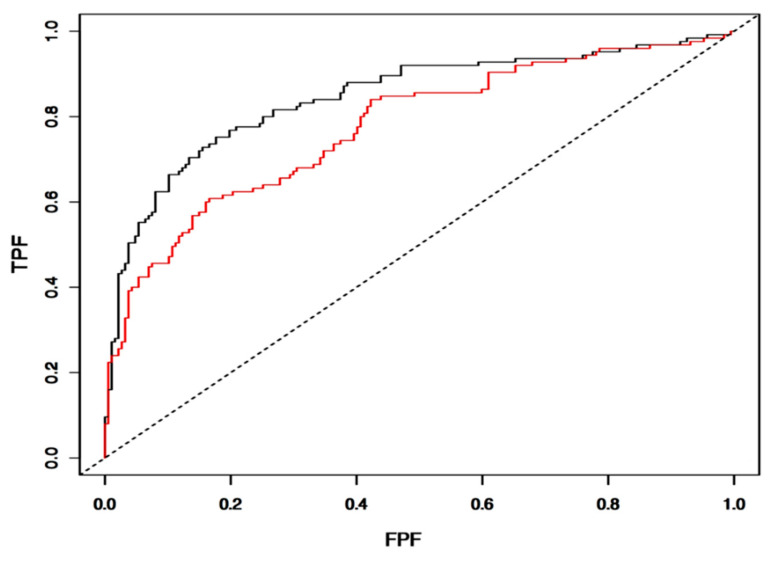
The figure above shows our ROC curve for IntegralVac’s performance accuracy for all overall predictions. The area under the curve quantifies the model’s ability to predict and sort epitope subunits with great accuracy; as a large area is seen in the figure, the data suggests that accuracy is relatively high. Predictions were generated for overall validation of IntegralVac by random selection from all peptide datasets, though still segregated by peptide type, i.e., viral vs. tumor data.

The diagnostic performance of the IntegralVac framework was evaluated using a receiver operating characteristic graph. A receiver operating characteristic (ROC) curve denotes a probability plot depicting the accuracy performance of a classifier model with varying discrimination thresholds. This was tested with IntegralVac’s test and train set, each containing 200–500 peptides randomly selected from the larger dataset. The threshold ranges from 0 to 1, with the “1” denoting 100% accuracy. Figure 7 shows the generated ROC curve for IntegralVac’s performance. The area under the ROC curve, or AUC, indicates accuracy as a numerical value for the test set. FPF represents false positives, i.e., data incorrectly marked as immunogenic, as a percentage, and is lower as the threshold decreases. The ROC plot indicates that IntegralVac has a high accuracy due to the large AUC for the tested datasets.

## 4. Discussion

Deep learning methods for predicting epitope binding are established to have high efficacy [7,8,9]. The primary aim of IntegralVac was to increase immunogenicity prediction accuracy compared to NetMHCPan through the integration of prior deep learning tools, thus providing an example for future research in integrating AI-based methods for peptide vaccine design. Our study analyzes a selection of clinically relevant deep learning models. It assimilates them to obtain data from various sources, including cancer and COVID data sourced from both IEDB and in-house generated data.

We referenced the DeepVacPred and MHCSeqNet models for examining viral data and, finally, integrated a hemolytic peptide design method to expand the coverage of our training model. Initially, the NetMHCPan server was used as base code to improve MHC Class I binding affinity predictions. We only used the Adam optimizer after we began integrating other models into the base code. We chose not to optimize only the individual models before combination, nor only the final, combined code. Instead, we found it more efficient to optimize each model individually after re-training, followed by repeated optimization after the combined models. In future iterations, we would continue to increase the coverage and complexity of the dataset by interspersing peptides between datasets with varying ratios from each source. Our datasets comprised epitopes length 8–10 obtained from mutated sequences from the IEDB’s NetMHCPan server. The top 27 [10] HLA alleles expressed in the human population, i.e., the IEDB HLA allele reference set with maximal population coverage, were selected to determine binding affinity with the control NetMHCPan server (Table 3). Using the methods from prior deep learning tools, we increased the prediction accuracy of the IEDB database’s mutated sequences.

In continuing to improve epitope prediction accuracy for non-viral data, i.e., cancer epitopes, we then used the MHCSeqNet and Hemolytic models. Along with amino acid sequence data for binding affinity predictions as represented by IC50 values, IntegralVac employed three other predictor designations, allergenicity, antigenicity, and toxicity, for DeepVacPred and in-house HRAS data. The prediction tools Vaxijen and AntigenPro were combined with IntegralVac to obtain allergenicity scores for the DeepVacPred dataset [7]. The overall antigenicity score for the DeepVacPred data was approximately 0.57 [7], a high antigenicity value, while the AllergenFP 1.0 server showed that allergenicity is negligible for the DeepVacPred data. These values suggest that the predicted epitopes can produce an immune response to the necessary antigens post-vaccination, as high antigenicity corresponds to a more robust antibody-binding capacity [13]. Following antigenicity and allergenicity predictor results, IntegralVac used the protein screening mode in the ToxinPred server to predict epitope toxicity values for both the DeepVacPred and our data using a max input length of 50 amino acids for consistency between all data points. The checkpoint filter predictions were successfully integrated into IntegralVac to have similar data to the other prediction servers.

These additional predictors helped us construct an efficient prediction model with existing deep learning neural networks. To further validate our method for CTL epitopes, we compared IntegralVac against NetMHCPan, aiming to optimize predictors through targeted selection for future data of peptide sequences and HLA molecules. As antigenic peptides were recognized and can elicit an immune response through cytotoxic B- or T-cell stimulation, lower immunogenicity scores were improved through machine learning algorithms instead of the more costly in vitro procedures, particularly because allergenic reactions and difficulties in clearance from the bloodstream are limited [14].

We were able to complete this process of expanding data coverage for IntegralVac by integrating the MHCSeqNet algorithm. The MHCSeqNet server aimed for universal MHC binding predictions for any MHC ligand peptidome dataset to be an accurate and valid screening tool for cancer vaccine development. We initially decided to utilize two different allele representations: one with a straightforward prediction server that did not cross-analyze MHC alleles and another based on amino acid positions in the peptide sequence that would allow for cross-processing of alleles. After integrating into our existing code, we decided to use the second framework, as cross-processing alleles allowed us to calculate immunogenicity scores for all 27 HLA Class I alleles [15]. To prevent the generation of opposite binding affinity classifications between different frameworks, we successfully removed low-confidence entries and proceeded with further optimization after integrating the Hemolytic model.

Our IntegralVac tool had the primary objective of achieving a more incredible speed and efficiency in vaccine design than other in silico methods. With IntegralVac, we propose a novel methodology to enhance the dataset coverage and range of existing in silico methods by combining them into a single comprehensive tool. We aimed to propose a pathway for future in silico tools to use a similar methodology for increased immunogenicity prediction accuracy. This is especially evident when the tool is compared with the Vaxijen server, which reaches an approximate 0.74 accuracy, at most, with a smaller dataset. This study and its referenced literature prove that deep learning methods can be utilized to model future options for viral vaccine design. Our study demonstrates that integrating different server methods is possible and can be performed more effectively for future in silico vaccine design. The large area under the returned ROC curve implies a very successful prediction model and can serve as a diagnostic for successful prediction models in the future.

**Figure 8:**
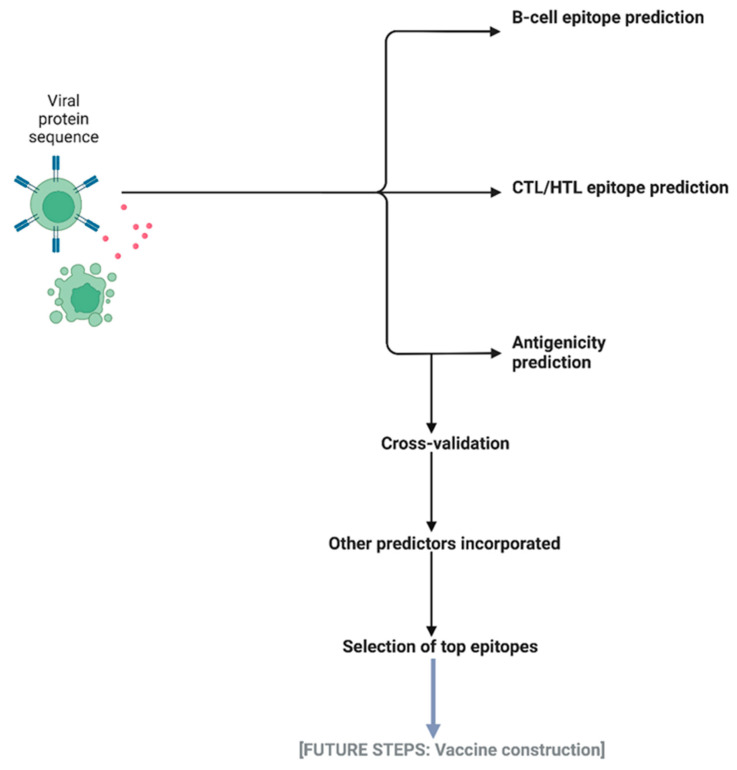
In using IntegralVac for eventual vaccine construction, we follow the same steps as our referenced models for the evaluation and selection of highly immunogenic peptides. This number should be greatly decreased (i.e., <30) from the dataset that the epitopes were selected from, as only the most highly antigenic and least allergenic epitopes would be utilized in a vaccine. Although our study investigated primarily T-cell epitopes, a fully comprehensive vaccine would select B-cell epitopes, as well, for maximal coverage.

With successful epitope prediction, we can proceed with improving the construction of a peptide vaccine in our next steps (Figure 8). By repurposing the cross-validation methods used for cleaning our datasets, IntegralVac can begin by removing the overlapping sequences between the top peptides to avoid autoimmune error interference. One method for doing so is through the usage of the BLASTp algorithm to compare local overlap between amino acid sequences of input proteins [7]. Other future steps we can take to construct a comprehensive peptide vaccine include expanding the coverage of IntegralVac to both B- and T-cell epitopes, thus increasing the number of potentially immunogenic peptides to select from. Thus, similar modes of integration, as done by IntegralVac for epitope prediction, can potentially be performed for incorporating epitopes within a vaccine, as well.

## 5. Conclusions

Among a myriad of vaccine design options, peptide vaccines hold invaluable potential due to being cost-effective and having an easily reproducible design [16]. In silico vaccine design is even more advantageous due to reducing waste and labor-intensiveness of the method relative to in vivo models. IntegralVac maps future deep learning possibilities in immune-informatics by integrating several in silico methods, including DeepVacPred, MHCSeqNet, and the hemolytic peptide vaccine design models. IntegralVac demonstrates the options for utilizing multiple deep learning models in conjunction to improve upon existing peptide binding prediction servers; these modifications included further rounds of optimization, binary classifier methods, and retraining methods.

IntegralVac is our comprehensive tool designed to increase accuracy for predicting immunogenicity and binding affinity of peptides sourced from tumor and COVID proteins. It was constructed by integrating three primary deep learning models: DeepVacPred, MHCSeqNet, and a non-hemolytic peptide prediction server. In referencing these tools, we were able to increase IntegralVac’s immunogenicity prediction accuracy, expand data coverage to multiple source data types, such as both viral and tumor peptides, increase binding affinity prediction accuracy and viable data inputs to both peptide sequences and amino acid properties, and develop better outlier detection methods. We hope that IntegralVac can illustrate the potential for AI-based peptide vaccine predictors with these advantages.

Therefore, our approach retains excellent potential for future study and expansion to a variety of sub-fields in oncology and immunology, i.e., using peptide and antigen characteristics to predict T-cell response. Much like wet-lab vaccine design, immunology assay processes remain expensive and lack concordance across many anti-reagent antibodies. Integration with gene expression is likewise possible. Thus, this work is foreseen to be valuable for MHC ligand prediction (potentially both Class I and Class II with time) and neoepitope screening for vaccine design, as well as training of binding and presentation models for T-cell-based immunotherapy and broader medical challenges essentially by “reprogramming” the systems that elicit an immune response in the body. Ultimately, this research ventures plausible methodologies for designing a reliable predictor tool. Further, this study opens pathways to using machine learning for vaccine design in the future, made possible by the promising work performed by the preceding researchers referenced in this study. The code for our study’s referenced methods is available for download in the Supplementary Materials section.

## 6. Limitations

We did not assess IntegralVac’s performance compared to the three other referenced methods, as their aims in generating predictions were different from ours. For example, we primarily assessed improvements in immunogenicity and binding affinity score predictions. In contrast, other models examined a range of other predictors and, thus, could not provide a valid comparison for prediction accuracy.

Several limitations remained upon integrating these models in our honing of prediction accuracy. Firstly, MHC Class II molecules’ prediction performance has been consistently limited across all training models. Class II molecules were unused for this integrated model due to a lack of concordance with training for Class I data. Likewise, there is a drawback of using cancer data in the data collection method itself, which is spectrometry. Spectrometry data are the primary dataset used to obtain tumor epitope sequences for in silico analysis. The human population has multiple MHC II molecules, and these spectrometry data pose difficulties in determining the same MHC molecule a peptide is bound to. This restricts the range of personalized vaccines generated and, therefore, creates difficulty in making large-scale deep learning-based predictions for larger datasets. With respect to dataset size, IntegralVac is currently limited to hosting several thousand peptides. One method to potentially increase this number is by developing IntegralVac’s compatibility with more HLA alleles instead of the 12 most common alleles within the population. By studying MHC entries consisting of more HLA-A and HLA-B alleles, as well as expanding coverage to HLA-C alleles, our laboratory may generalize our model to far larger datasets. Thus, rather than being a finalized prediction tool, IntegralVac acts as a hypothesis to answer whether deep learning tools can genuinely be utilized and merged for multi-epitope vaccine design.

## Figures and Tables

**Table 1:** The table depicts the top five CTL epitope predictions referenced from DeepVacPred data, with allergenicity, antigenicity, and toxicity filters. AllergenFP did not predict allergenicity values, and ToxinPred did not predict toxicity values for any sequences. This is evidence of the top epitopes not exhibiting significant levels of allergenicity or toxicity. IntegralVac’s results matched precisely with the DeepVacPred server’s predictions using the same checkpoint filter prediction servers despite modifications compared to NetMHCPan. This similarity in results was observed despite IntegralVac’s use of other deep learning tools to improve the accuracy of predictions. Thus, we can infer that these five epitopes are viable vaccine subunit candidates for each checkpoint filter criteria (i.e., allergenicity, antigenicity, and toxicity).

Peptide Sequence	DeepVacPred Subunit	Vaxijen	AntigenPro	AllergenFP	ToxinPred
FVFKNIDGYFKIYSKHTPINLVRDLPQGFS	5	0.476	0.477	-	-
LGQSKRVDFCGKGYHLMSFPQSAPHGVVFL	23	0.671	0.736	-	-
LGVYYHKNNKSWMESEFRVYSSANNCTFEY	4	0.390	0.736	-	-
ILDITPCSFGGVSVITPGTNTSNQVAVLYQ	13	0.832	0.403	-	-
LPDPSKPSKRSFIEDLLFNKVTLADAGFIK	19	0.361	0.499	-	-

**Table 2:** The accuracy and AUC of IntegralVac’s predictions. The AUC and accuracy values were greater than those of the control methods but remained lower than the AUC/accuracy of the referenced methods, such as DeepVacPred and MHCSeqNet. This may be because IntegralVac increased the coverage and complexity of the dataset and used multiple sources for our peptide data, thus affecting sensitivity and specificity values and lowering accuracy.

Validation	AUC	Threshold	Accuracy	Sensitivity	Specificity
Train set	0.9608	0.32	0.960	0.95	0.95
Test set	0.9046	0.5	0.90	0.90	0.90

**Table 3:** The table depicts the top 27 HLA alleles in the human population.

Top 27 Population Alleles from which Epitopes Are Extracted	
HLA-A	HLA-B
HLA-A*01:01	HLA-B*07:02
HLA-A*02:01	HLA-B*08:01
HLA-A*02:03	HLA-B*15:01
HLA-A*02:06	HLA-B*35:01
HLA-A*03:01	HLA-B*40:01
HLA-A*11:01	HLA-B*44:02
HLA-A*23:01	HLA-B*44:03
HLA-A*24:02	HLA-B*51:01
HLA-A*26:01	HLA-B*53:01
HLA-A*30:01	HLA-B*57:01
HLA-A*30:02	HLA-B*58:01
HLA-A*31:01	
HLA-A*32:01	
HLA-A*33:01	
HLA-A*68:01	
HLA-A*68:02	

Within the human leukocyte antigen (HLA) complex, HLA-A and HLA-B are two closely related proteins significant for the cytotoxic t-cell controlled immune response to pathogens. In total, 27 alleles were chosen to maximize possible data coverage worldwide [10]. “HLA-A” and “HLA-B” are the antigen serotype groups while the numbers following the asterisk (*) describe the specific serotype allele, or variation within that group of antigens. From these 27 expressed alleles, NetMHCPan and IntegralVac return epitopes and their predicted metrics, i.e., immunogenicity score and binding affinity value. These metrics are denoted by a percentile ranking and value describing IC50.

## Data Availability

Figure 6 and Figure 8 created with BioRender. Datasets and supporting results can be found in the Supplementary Tables, while the base code and referenced datasets were taken from three sources under an Open Access/permissions license as sourced via a hyperlink to its Creative Commons CC BY License: (1) An in silico deep learning approach to multi-epitope vaccine design: a SARS-CoV-2 case study [Creative Commons CC BY license]. (2) MHCSeqNet: a deep neural network model for universal MHC binding prediction [Creative Commons CC BY License]. (3) Machine learning-guided discovery and design of non-hemolytic pep-tides [Creative Commons CC BY License].

## Supplementary Materials

The following supporting information can be downloaded at: https://www.mdpi.com/article/10.3390/vaccines10101678/s1, Table S1: IntegralVac made predictions for KRAS epitopes with the same relative accuracy as the control IEDB method for the selected G12C mutation in the Ras proto-oncogene; Table S2: We selected 16 top peptides expressing the nine unique mutations in the HRAS gene. Each peptide binds multiple HLA alleles, and we predicted a more significant number of peptides than the KRAS epitopes but with the same relative diversity; Table S3: Nsp12 is the first of two non-structural proteins we analyzed from coronavirus. We selected CD8 epitopes to be tested with Integral-Vac and compared them to IEDB methods for epitope prediction; Table S4: We obtained nsp3 epitopes to investigate coronavirus epitope vaccine design as it is the largest multi-domain protein produced by the virus. Due to its large size, we were able to use the greatest number of experimental epitopes for validation and predicted CD8 epitopes with affinity to murine alleles; Table S5: In our laboratory, we previously identified ORF3a as one of two identified potential vaccine targets against SARS-CoV-2, specific to the South African and Indian variants. Although Parn et al. isolated both CD8 and CD4 epitopes to construct a multivalent epitope, we used CD8 epitopes only and obtained similar overall rankings to IEDB predictions [11]; Table S6: For ORF9b data, our epitopes were obtained from the Brazilian, Indian, South African, and UK variants, and we selected epitopes with an immunogenicity score > 0 for further analysis. Integral-Vac was able to predict immunogenic epitopes with high accuracy, as verified by our control in-house predictions made with the IEDB server; Table S7: The nucleocapsid N protein is one of the four main structural proteins encoded within the SARS-CoV-2 genome. Our laboratory had decided to investigate N proteins as N protein. T-cell epitopes have a longevity of up to 17 years, longer than the spike (S) protein. Yet, many SARS-CoV-2 vaccine construction strategies recruit only S proteins in the field. We were able to present high-affinity CD8 epitopes for specific antigen targets; Table S8: Like N proteins, spike S proteins are major antigens in SARS-CoV-2 [17]. Our laboratory thus found S proteins to be a prime target for determining immunogenicity. Population coverage is lower for N and S proteins than for the non-structural proteins our laboratory had isolated [11]. However, we could isolate highly immunogenic N protein epitopes and categorize them by binding score rank; Table S9: We used this dataset as our initial control before running Integral-Vac on our in-house laboratory datasets. These data comprised MHC I peptides obtained from the IEDB database and were previously tested by the DeepVacPred server. Integral-vac was able to produce similar binding score predictions and validate our method; File S10: Referenced code of DeepVacPred method integration; File S11: Referenced code of MHCSeqNet method integration.
